# Graphitic Carbon Nitride: Synthesis and Characterization, Monolayer at the Air–Water Interface, Langmuir–Blodgett Films, and Its Photocatalytic Performance

**DOI:** 10.1021/acsomega.5c02295

**Published:** 2025-04-15

**Authors:** Éverton Wilker A. Almeida, Claire M. C. Dazon, Mariandry D. V. R. Rodriguez, Thatyane M. Nobre, Márcio César Pereira, Douglas S. Monteiro

**Affiliations:** † Institute of Science, Engineering and Technology, Federal University of Jequitinhonha and Mucuri Valleys, Teófilo Otoni, Minas Gerais 39803-371, Brazil; ‡ Institute of Physics of São Carlos, University of São Paulo, São Carlos, São Paulo 13560-970, Brazil

## Abstract

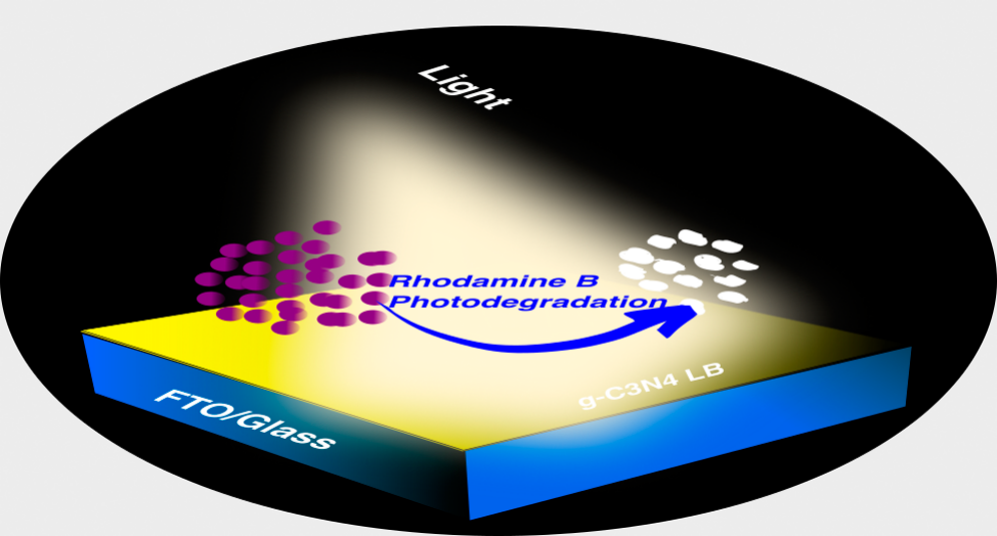

Langmuir–Blodgett technique (LB) is a powerful tool for ultrathin film fabrication. LB film production has attracted much attention since film thickness and architecture can be controlled at the molecular and atomic levels. However, a lack of studies still exists regarding LB films of nanomaterials, especially 2D materials. In this context, the present work aims to produce and characterize LB films of g-C_3_N_4_, a layered nonmetallic photocatalyst. For this purpose, g-C_3_N_4_ was synthesized, exfoliated, and characterized by vibrational spectroscopy, X-ray diffraction, morphological analysis, surface area determination, hydrodynamic radius, and zeta potential. Before LB film preparation, experiments of g-C_3_N_4_ Langmuir films at the air–liquid interface were performed. Surface pressure, Brewster angle microscopy, and surface potential experiments of Langmuir films reveal their properties and an ideal condition for monolayer transfer for solid substrates. LB films were transferred to silicon and FTO-coated glass with the latter showing excellent coverage, making it the substrate of choice for photocatalytic assays. The g-C_3_N_4_, in both powder form and as an LB film, achieved degradation rates of 96.3% and 73%, respectively, of the rhodamine B present in the medium after 8 h of reaction. After 24 h, the reused LB film maintained its photocatalytic activity, continuing to degrade 73% of the probe molecule, demonstrating that the transferred material adheres well to the substrate surface. These results present promising opportunities for applying g-C_3_N_4_ LB films in photocatalytic, photovoltaic, and other chemical conversion devices.

## 1 Introduction

Photocatalytic or photovoltaic systems frequently require the production of thin films of nanomaterials. Concerning metallic oxides and 2D materials as semiconductors, drop-casting, spin coating, slot-die coating, or doctor blade coatings are traditionally used for film preparation. To explore the full potential of nanomaterials, the control of film thickness, interlayer configuration, and composition is of great importance. In this sense, the Langmuir–Blodgett (LB) technique is a relevant tool for producing thin films even down to the atomic scale. 2D materials LB films have gained attention since they combine nanomaterial’s unique properties and the LB technique’s versatility. Graphene oxide,^1^ graphene,^2^ and molybdenum sulfide,^3^ as 2D nanomaterial examples, were studied in their monolayer form at the air–liquid interface and LB film. These studies involved surface chemistry analysis, including typical surface pressure (π) versus area isotherms and LB film morphology.

On the other hand, graphitic carbon nitride (g-C_3_N_4_) is a semiconductor that has been extensively studied in recent years. It has been used in water splitting,^4^ CO_2_ reduction,^5^ hydrogen production,^6^ or organic dye degradation^7^ process. g-C_3_N_4_ is an entirely metal-free photocatalyst, and its synthesis requires easy-to-find precursors such as urea^8^ and melamine,^9^ and ultrasound exfoliation can produce sheets of atomic thickness.

Despite this, controlled transfer of g-C_3_N_4_ LB films is yet to be demonstrated. Langmuir films are prepared by spreading amphiphilic material solutions at the air–water interface. Generally, nonpolar solvents that perfectly wet subphase surfaces are ideal for amphiphilic solution preparation with a concentration in the range of 1 μM. A typical spread procedure lets a drop formed in a needle tip touch the water surface. Using only a few tens of microliters of the solution allowed the formation of a monolayer at the interface as the nonpolar solvent evaporated. Unlike ordinary amphiphiles (such as phospholipids and fatty acids), nanomaterials do not dissolve in nonpolar volatile solvents. Dispersions in polar solvents are often used instead of a solution for Langmuir monolayer studies using metallic oxide nanoparticles or 2D nanomaterials. Since polar solvents such as methanol or ethanol are water-soluble, preparing the Langmuir film of nanomaterials using these solvents may require a large amount of dispersion volume in the polar solvent. As the dispersion drops, it leads to the loss of most of the spreading materials carried by the drop that will submerge and directly mix with water, impeding the Langmuir film formation.

The electron-spray spreading solves a common problem for preparing Langmuir monolayers of nanomaterials that generally form stable dispersion in polar solvents.^10^ In this technique, material loss through intermixing with the subphase is circumvented by the direct spreading of tiny droplets of the dispersion that can reach the order size micron to submicron scale.^11,12^ The droplet size can be smaller as solvents evaporate, avoiding losing material to the bulk subphase and favoring Langmuir film formation. Even spreading large volumes of nanomaterial dispersion by the conventional method did not promote formation of the Langmuir film. On the other hand, small dispersion volumes are sufficient to form a stable monolayer using the electron-spray technique.

Most efforts to study materials such as Langmuir monolayers typically focus on amphiphilic organic molecules, such as fatty acids and phospholipids. To the best of our knowledge, this is the first time that studies on the behavior of g-C_3_N_4_ at the air–liquid interface in the form of a Langmuir film (insoluble monolayer) have been presented in the literature. Furthermore, there are no existing records of studies on the photocatalytic performance of a 2D nanomaterial in the form of an ultrathin LB film.

So, this work shows an easy method for LB thin film transfer of g-C_3_N_4_ and proposes the investigation of its photocatalytic properties. For this purpose, g-C_3_N_4_ was synthesized from urea and characterized to prepare the nanomaterial floating layers. The surface chemistry properties were also verified to obtain excellent experimental conditions to promote LB film transfer. Then, the LB g-C_3_N_4_ films on silicon and fluorine-doped tin oxide (FTO) glass morphology were investigated and compared to get the best condition for testing photocatalytic performance using the rhodamine B (Rh B) as a probe molecule.

## 2 Experimental Section

### 2.1 Materials

Ultrapure water (18.2 MΩ·cm at 25 °C, pH 6.5) was obtained by tap water purification on a DirectQ 3UV system (Merck Millipore). Isopropyl alcohol (Neon) and urea (Sinthy Chemicals) were ACS grade. Rh B (Isofar) was 99.75% and P.A. grade. FTO and silicon substrates were purchased from Sigma-Aldrich.

### 2.2 g-C_3_N_4_ Synthesis and Characterization

g-C_3_N_4_ particles were synthesized as described by Liu et al.,^13^ with minor modifications. 10 g of urea was heated at 550 °C for 3 h under ambient pressure on a muffle furnace. After being cooled, the pale-yellow product was washed three times with ultrapure water, rinsed with isopropyl alcohol, and dried at 80 °C for 24 h. Then, the g-C_3_N_4_ powder was exfoliated to produce C_3_N_4_ nanosheets. For that, g-C_3_N_4_ was calcined at 550 °C for 2 h to produce oxidized g-C_3_N_4_, dispersed in isopropyl alcohol (0.1 mg mL^–1^), and sonicated for 4 h. The synthesis yield was 4.5 wt %.

The exfoliated g-C_3_N_4_ structural analysis was carried out using an X-ray diffractometer (Shimadzu XRD-6000), and chemical functional groups were analyzed by attenuated total reflectance with Fourier-transform infrared spectroscopy (ATR/FTIR) (Frontier, PerkinElmer). Morphological analyses were carried out with a scanning electron microscope (SEM) (VEGA 3 LMU, Tescan) (exfoliated and bulk g-C_3_N_4_) and transmission electron microscope (TEM) (JEM2100 LaB6, Jeol) (exfoliated g-C_3_N_4_). A zeta potential analyzer determined the hydrodynamic radii and zeta potential of exfoliated g-C_3_N_4_ nanosheets in ultrapure water (Zetasizer Nano, Malvern).

### 2.3 Langmuir Monolayers and Langmuir–Blodgett Film Transfer

Langmuir monolayer experiments were performed in a Langmuir trough (KN2002, KSV Nima) with dimensions of 100 mm × 250 mm and 180 mL. Dispersions of exfoliated g-C_3_N_4_ in isopropyl alcohol (6 mL of a 7.5 mg mL^–1^) were spread at the air–liquid interface using an electrospray system, as introduced by Huang and co-workers with modifications.^10^ For this purpose, a glass syringe was placed 2.5 cm over the Langmuir trough. The high-voltage power supply (QSC 0630, Inergiae) was connected to a steel needle, and a grounding wire was immersed in the water subphase. A vessel placed 35 cm above the trough was used to store g-C_3_N_4_ dispersion and fill the syringe by gravity. The liquid flow was controlled by adjusting a roller clamp coupled to the connecting tube between the syringe and the vessel. The electric field was set to 13 kV, and the electron spray spreading rate was adjusted to 40 mL h^–1^. Langmuir film compression isopropyl alcohol was tested by spreading the same volume for exfoliated g-C_3_N_4_ dispersions. It did not alter the water surface pressure after a complete barrier compressing cycle, indicating the absence of surface-active impurities in the solvent. Surface pressure (π) versus trough area (*A*) isotherms were recorded at a compression rate of 10 mm mim^–1^. Also, Langmuir films at the air–water interface were characterized using a surface potential sensor (SPOT, KSV Nima) to obtain surface potential versus surface trough area (*A*) isotherm. For this purpose, the surface potential of the monolayer was set to zero after spreading the g-C_3_N_4_. Monolayer morphological structure and homogeneity were analyzed by a Brewster angle microscopy (MicroBAM, KSV Nima). LB films were prepared by upstroking the substrate (glass or silicon) through the Langmuir film at a 5 mm min^–1^ rising rate. LB films morphology was characterized by SEM.

### 2.4 Photocatalytic Performance of g-C_3_N_4_ as Powder Dispersions and as Langmuir–Blodgett Films

The exfoliated g-C_3_N_4_ heterogeneous photocatalytic activity assays, as powder dispersions or LB thin films, were carried out considering the previous paper presented by Pirard et al.^14^ The reactions were conducted in an entirely light-sealed environment, with the temperature controlled at 23 °C using a thermostatic bath (MR F25, Julabo). On a Petri dish, an LB film of g-C_3_N_4_ was placed in contact with 25 mL of an Rh B solution (10 mg L^–1^) under illumination from a xenon visible light source (Xenolux 300, Confiance Medical) with a 300 W lamp power at 100% intensity through an optical fiber cable positioned 1.5 cm above the LB films. The same height was used for experiments without the film. The solution was kept under agitation using a magnetic stirrer and a Teflon-capped magnetic bar for 8 h. Every half hour, 200 μL aliquots of the Rh B solution were taken and transferred to a quartz cuvette, where the volume was completed with ultrapure water up to 500 μL. The concentrations of Rh B in the samples were determined by comparing the analyte’s absorption value to a standard calibration curve obtained through UV–vis spectrophotometry (WUV-M51, Weblabor) with a wavelength scan in the range of 800–200 nm. For comparison purposes, blanks were obtained using the Rh B solution in contact with (i) 0.0003 g of the dispersed powder material (corresponding to the amount of material contained in a film plate produced); (ii) the plate with the LB film of g-C_3_N_4_ in the absence of light; (iii) the plate with the g-C_3_N_4_ film in the presence of light; and (iv) the reuse of the plate with the film, where it was washed with ultrapure water and allowed to dry for at least 24 h at room temperature.

## 3 Results and Discussion

### 3.1 g-C_3_N_4_ Characterization

The methodology used in this work made it possible to achieve a yield of 4.5% for the thermal decomposition reaction of urea and the formation of g-C_3_N_4_. This yield is consistent with values between 3 and 6% found in the literature.^13,15^

**Figure 1:**
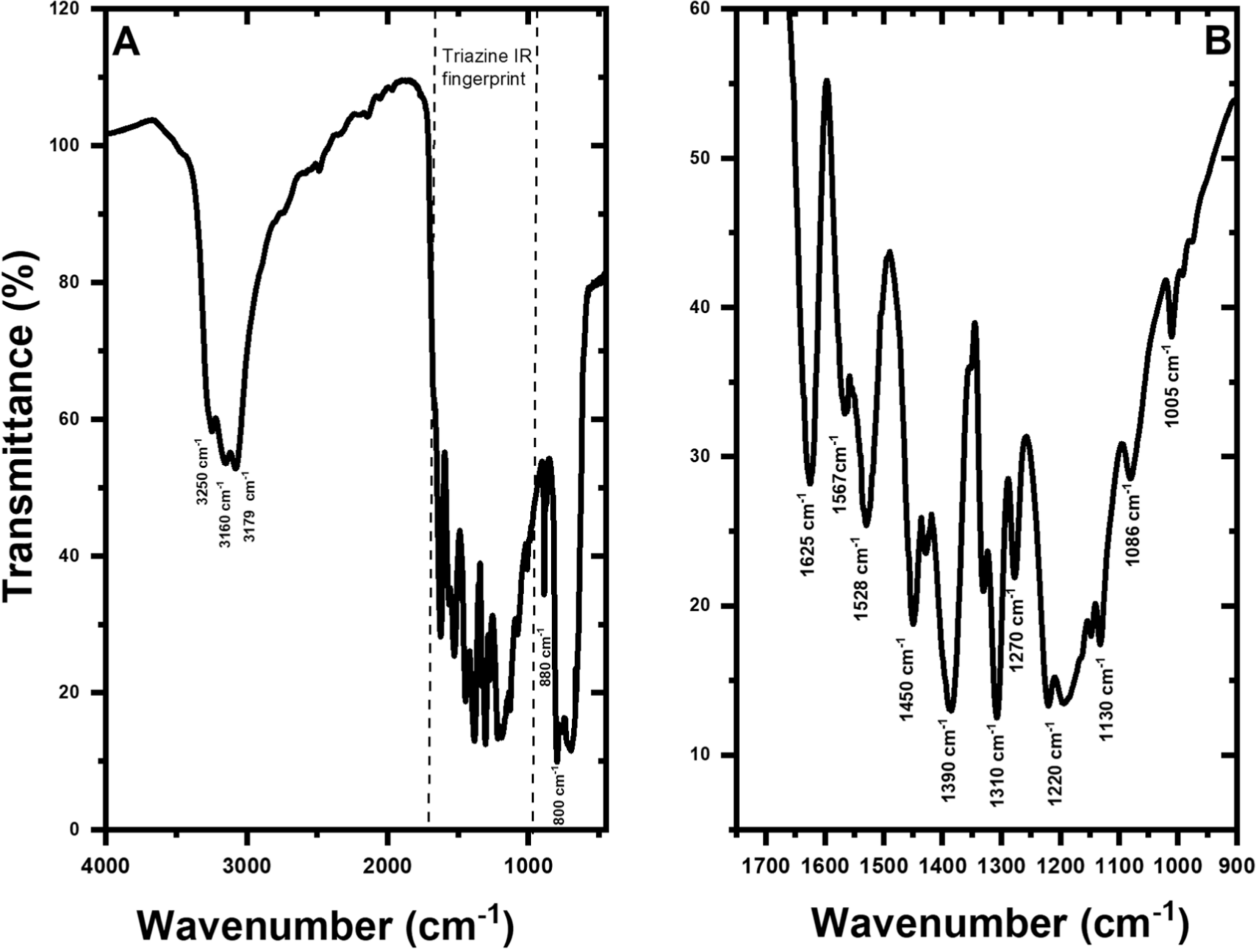
Infrared spectra of g-C_3_N_4_ (A) and triazine infrared fingerprint region (B).

The chemical structure of exfoliated g-C_3_N_4_ was investigated by FTIR (Figure 1A,B). Bands in the region of 3000–3500 cm^–1^ are attributed to stretching vibration of the N–H bond in the C–N(H)–C of heterocycle and C–NH_2_ terminal groups of g-C_3_N_4_ layers^16^ and O–H bond stretching of water molecules adsorbed on the material surface.^17,18^ Other typical vibrational absorption bands of g-C_3_N_4_ appear on the fingerprint region of tris-*s*-triazine (Figure 1B), ranging from 1000 to 1640 cm^–1^. The sharp and strong band at 1625 cm^–1^ is due to the in-plane deformation of NH_2_. The bands at 1567, 1528, 1450, and 1390 cm^–1^ are due to the stretching of C=N and C–N bonds of secondary and tertiary aromatic amines.^15,19,20^ The infrared absorption due to the in-plane deformation of NH and the stretching of CN bonds correspond to a coupled band at 1310 cm^–1^. The bands at 1270 cm^–1^ and 1220 cm^–1^ correspond to the stretching of C–N or NH bonds. The bands at 1130 and 1086 cm^–1^ are attributed to the rocking vibration of hydrogen atoms on NH_2_ groups, and the weak band at 1010 cm^–1^ is due to aromatic ring stretching. In the lower frequency region of the FTIR spectrum, the sharp band around 880 cm^–1^ is related to the stretching of the N–H bond of amino groups.^16^ In contrast, the band at about 800 cm^–1^ can be attributed to the out-of-plane bending vibration of the cyameluric nucleus (C_6_N_7_) in heptazine rings.^18,21^ The characteristic bands of g-C_3_N_4_ identified in the FITR spectrum indicate that the urea precursor was successfully polymerized into g-C_3_N_4_.

**Figure 2:**
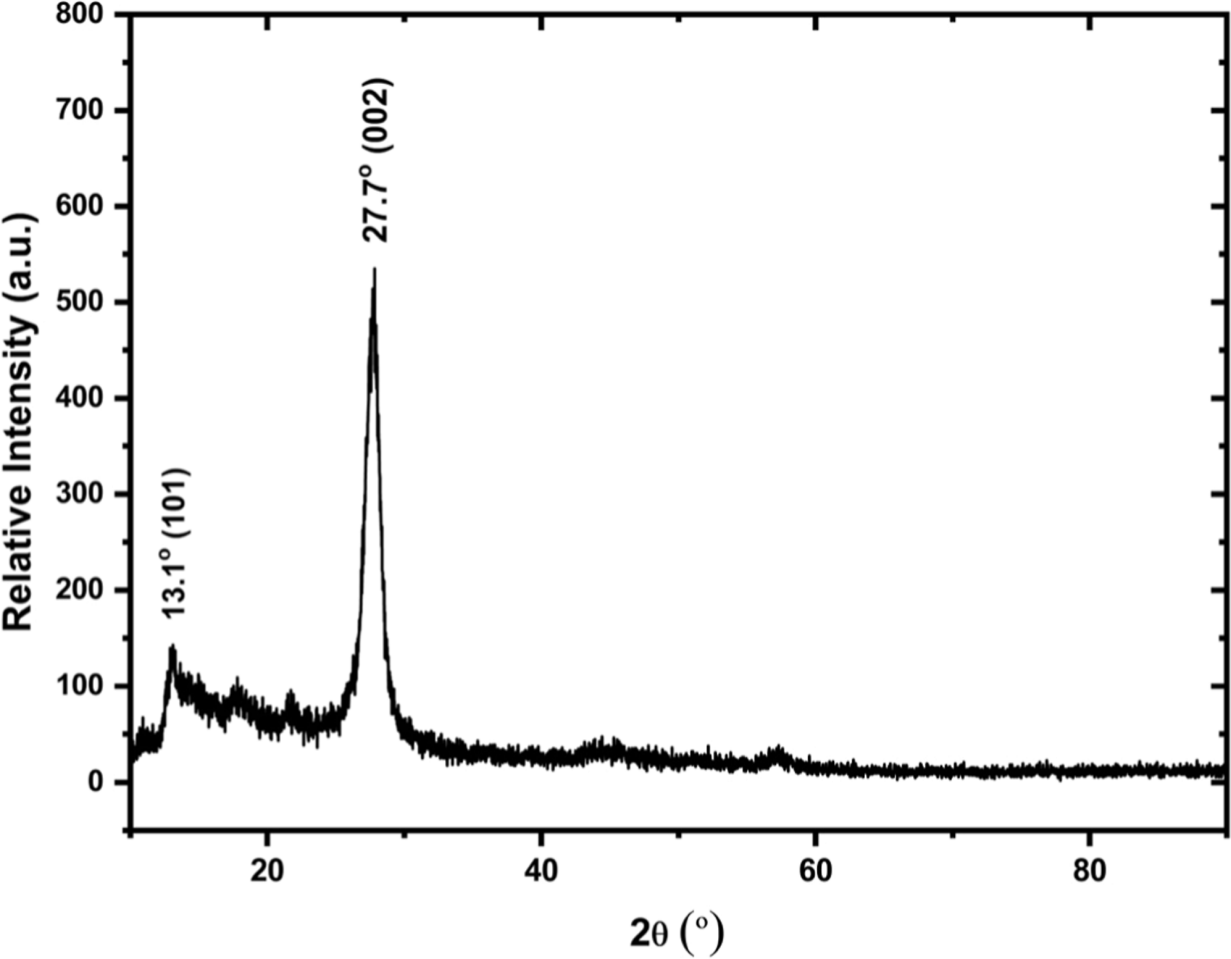
X-ray diffraction pattern of g-C_3_N_4_ powder.

The X-ray diffraction pattern (Figure 2) was used to identify the material crystal structure. It reveals two major diffraction peaks around 13.1 and 27.7°, characteristics of heptazine-based samples synthesized above 500 °C.^22,23^ The strong peak at around 27.7° is attributed to the (002) diffraction plane with stacking aromatic rings and interplanar distance (*d*) of 0.325 nm.^24^ The weak peak at 2θ = 13.1° is assigned to the (100) diffraction plane and corresponds to the in-planar structural packing of tricycle tris-*s*-triazine units with *d* = 0.681 nm.^24−26^ This distance is smaller than the tris-*s*-triazine unit (ca. 0.73 nm), presumably due to the bending of 2D structures of the ring layers.^27^ In addition, there are also very weak diffraction peaks at 2θ between 14 and 25°, which corresponds to repeating planes in the layered structure of g-C_3_N_4_.^28^

**Figure 3:**
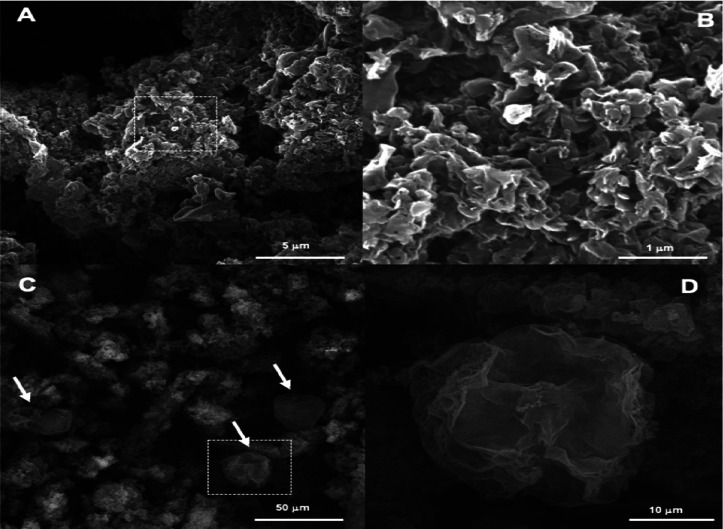
SEM images of (a,b) pristine and (c,d) exfoliated g-C_3_N_4_.

**Figure 4:**
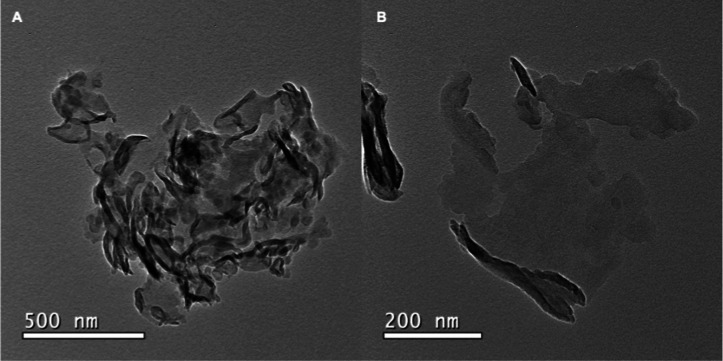
TEM images (a,b) of exfoliated g-C_3_N_4_.

The morphology and microstructure of the g-C_3_N_4_ samples are revealed by SEM (Figure 3) and TEM images (Figure 4). Dashed squares amplify the image area in Figure 3A,C, resulting in Figure 3B,D, respectively. A notable difference in the morphology of the samples is observed when images of the pristine (A) and exfoliated g-C_3_N_4_ are compared (C). Figure 3A shows clusters of agglomerated g-C_3_N_4_ particles of several huddled stacked sheets of g-C_3_N_4_. Exfoliation of g-C_3_N_4_ produced large smooth sheets with a width of about 26 μm as indicated by the arrows in Figure 3C and enlarged in Figure 3D.

TEM images of exfoliated g-C_3_N_4_ (Figure 4A,B) show sheets with roled-up edges about 400 nm in width. SEM and TEM images evidence that synthesized g-C_3_N_4_ has a layered structure that could be exfoliated into isolated single sheets.

The BET surface area of the exfoliated g-C_3_N_4_ sheets was 92 m^2^ g^–1^, which is about twice the surface area reported by Zhu et al.^29^ (40 m^2^ g^–1^) and Dong et al.^30^ (46 m^2^ g^–1^) for g-C_3_N_4_ produced from urea precursor. This significant difference in BET area indicates that the sonication step is essential to produce more isolated layers of g-C_3_N_4_, resulting in sheets with a higher surface area. The hydrodynamic radius of the exfoliated g-C_3_N_4_ particles determined by DLS measurements was ∼620 nm, which is consistent with the sheet size observed in the TEM images (Figure 4) concerning the higher value can be attributed to the fact that nanomaterials can aggregate in solution.^31^ The g-C_3_N_4_ zeta potential measured was −29.4 mV, close to the values reported in ultrapure water at pH 6.7.^30,32^ Above their point of zero charge (pzc = 5.1 in pure water),^29^ hydroxyl ions, generated by water autolysis, deprotonate primary and secondary amines in the g-C_3_N_4_ molecular structure. As a result, the predominant electric charge on the surface of g-C_3_N_4_ must be negative at pH 7.

### 3.2 g-C_3_N_4_ at the Air–Liquid Interface, Monolayer Properties, and LB Film Fabrication

**Figure 5:**
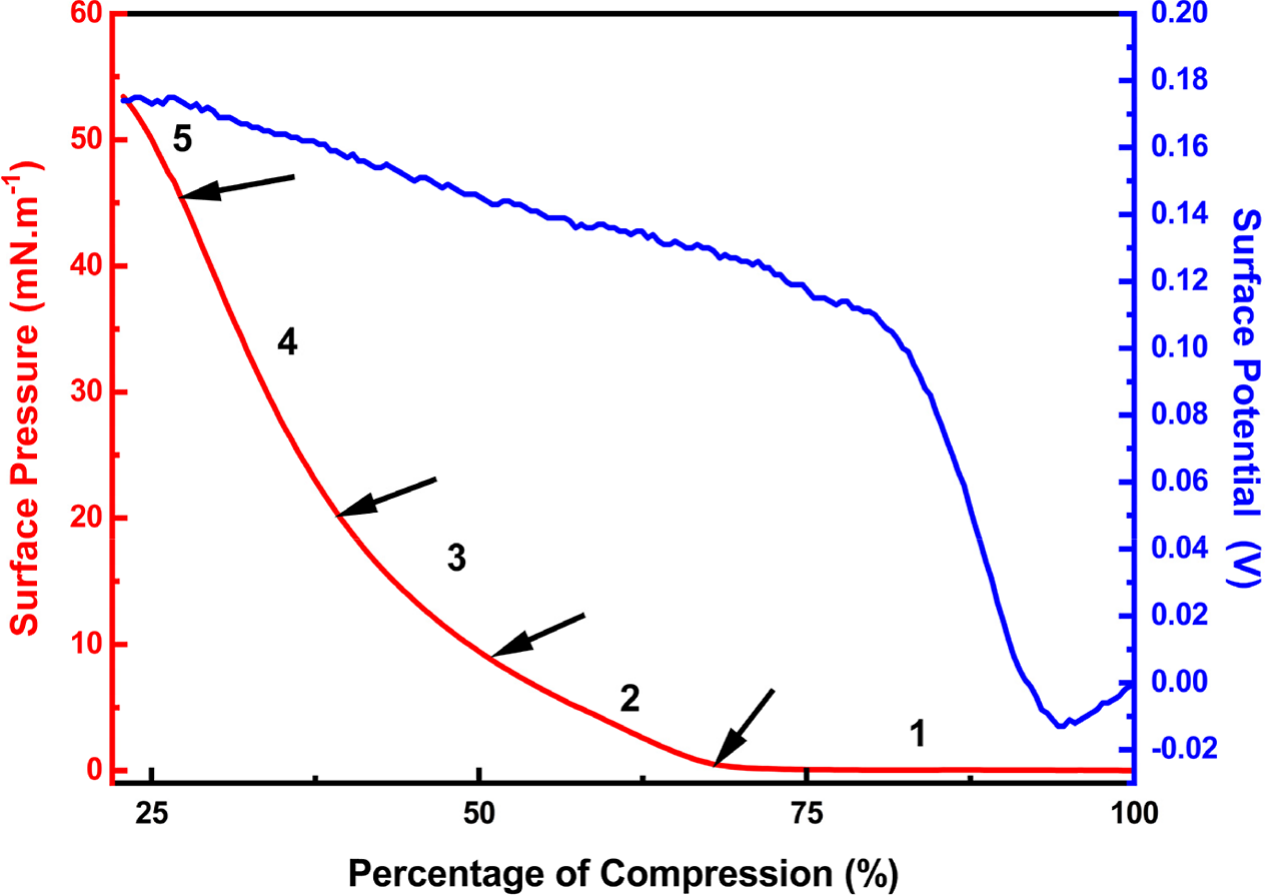
Air–liquid properties of the g-C_3_N_4_ Langmuir film: π × *A* (red line) and *V* × *A* (blue line) isotherms.

**Figure 6:**
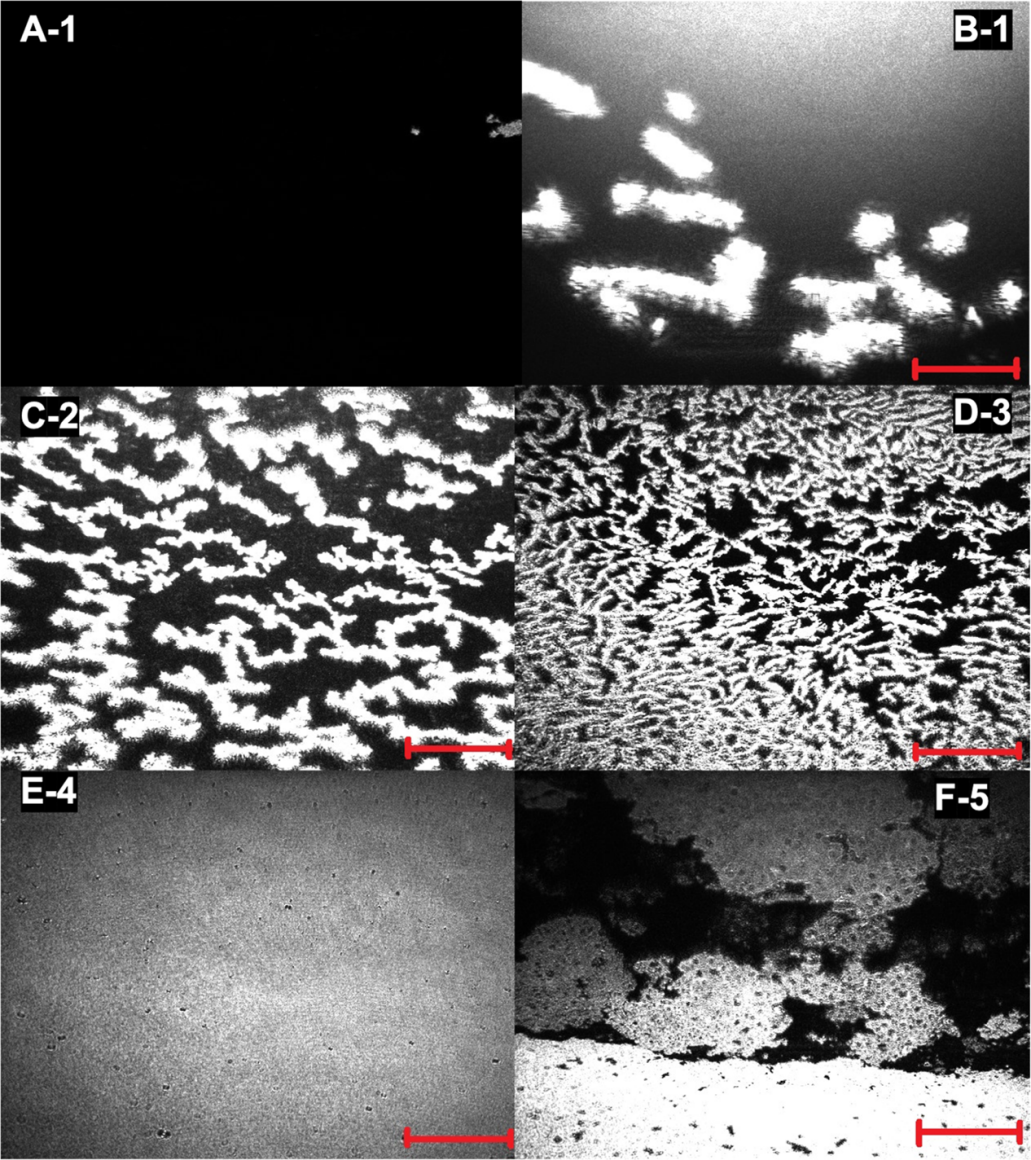
BAM images of g-C_3_N_4_ Langmuir monolayer at different surface pressure ranges: π < 1 mN m^–1^(A,B), ∼1 mN m^–1^ < π < ∼10 mN m^–1^ (C), ∼10 mN m^–1^ < π < ∼20 mN m^–1^ (D), ∼20 mN m^–1^ < π < ∼47 mN m^–1^ (E), and π > ∼47 mN m^–1^ (F). The Arabic numerals indicate the π range correspondent appointed on the π–*A* isotherm (Figure 5). The bar scale is 690 μm wide.

Figure 5 shows the π × *A* isotherm, and Figure 6 shows the BAM images of the g-C_3_N_4_ Langmuir film. In Figure 5, arrows are close to the surface pressure range where the observed significant morphology change of domain structures occurs, denoting the five phases of the Langmuir film indicated by numerals (also denoted in Figure 6). In the π × *A* isotherm, a large gas phase is observed, and a significant increase of surface pressure (π ≥ 1 mN m^–1^) only occurs when the barriers have already swept about 70% of the trough area (at ∼170 cm^2^). At this compression stage, very few floating and bright islands must correspond to aggregates of g-C_3_N_4_ on the surface, referring to the gas phase of the monolayer (Figure 6A,B). Next, as the barriers advance, it promotes an increase in π, and in the range of ∼1 and 10 mN m^–1^, it is possible to observe the formation of structures distinct from the gas phase (Figure 6C). There are floating cloud-shaped aggregates with rounded edges with a width of 59 ± 25 μm and a length of around 600 ± 208 μm. A shape and size change was observed on floating aggregates from ∼10 to ∼20 mN m^–1^ (Figure 6D). They acquire the shape of a less rounded edge, and the width decreases to 45 ± 12 μm and the length down to 255 ± 93 μm. The separated aggregates disappear from ∼20 mN m^–1^, forming a smooth domain with well-distributed pinholes (Figure 6E). This homogeneous domain consists of a dense g-C_3_N_4_ layer continuous phase, which is observed on the whole field of view of the microscope (3.6 × 4 mm). The typical collapse of phospholipids^33^ and fatty acids^34^ Langmuir monolayers, where an abrupt drop on π occurs, was not observed in the g-C_3_N_4_ recorded π–*A* curve. Instead, BAM analysis could confirm that additional compression after ∼47 mN m^–1^ promotes g-C_3_N_4_ Langmuir film collapse. The film structure cracks and separated plates of the g-C_3_N_4_ aggregates are observed. Figure 6F shows that these plates are submerged in the subsurface, creating a second layer film structure.

Simultaneously, the *V* × *A* isotherm of g-C_3_N_4_ Langmuir film can bring complementary insight into the above-mentioned film structure (Figure 5, blue line). As the *V* is proportional to the dipole moment, it changes as the film surface density increases under compression.^35,36^ Also, the van der Waals interactions can promote molecular reorientation and fold changes on the g-C_3_N_4_ sheets, thus contributing to variations in the dipole moment.^37^ When the barrier starts to compress, the surface potential is reduced from zero to a minimum of ∼−13 mV. At this stage, ample space between the g-C_3_N_4_ sheets and attractive long-range van der Waals forces predominate. Thus, the polarity of the C–N and N–H bonds in the g-C_3_N_4_ structure contributes to the formation of the first aggregates at the liquid–air interface. After the curve minimum point, a linear rise of *V* is observed as the barriers force the approach of g-C_3_N_4_ sheets, and film surface density increases. When the barriers have already swept ∼23% of the surface area (at *A* = 190 cm^2^), a change occurs on the *V* curve inclination. At this point, repulsive interactions between negatively charged sheets probably promoted significant molecular orientation and folding changes at the air–liquid interface. So, the *V* increases until the barriers stop, without another change in the curve slope, although the BAM experiment revealed g-C_3_N_4_ film collapse.

### 3.3 g-C_3_N_4_ Langmuir–Blodgett Films

**Figure 7:**
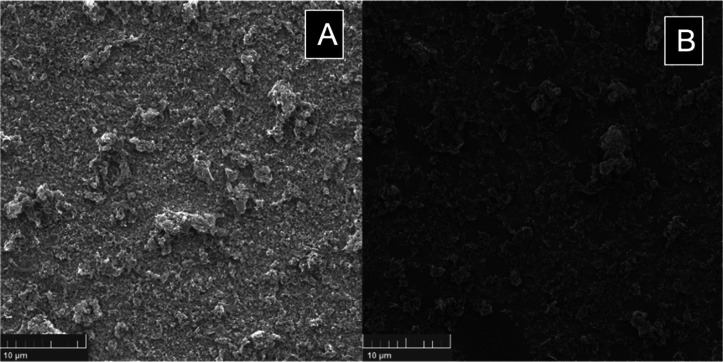
SEM images of one monolayer g-C_3_N_4_ LB transferred at 25 mN m^–1^ on (A) FTO-coated glass and (B) silicon substrates.

After studying the g-C_3_N_4_ Langmuir film structure, we prepared the LB films of the g-C_3_N_4_ monolayer. Figure 7 shows SEM images of LB films transferred at a π = 25 mN m^–1^ on (A) glass and (B) silicon substrates at the same magnification. At this surface pressure, the BAM images showed a dense, continuous, and homogeneous g-C_3_N_4_, which could provide ideal conditions for maximum substrate coverage. FTO-coated glass and silicon substrates were chosen because they are commonly used in photoelectrochemical and photovoltaic devices.

The reproducibility of the film characteristics was checked by performing two different LB transfer experiments. The g-C_3_N_4_ transference rates (TR) of the transfer processes were 0.9 and 0.8 for FTO glass and silicon, respectively. As the FTO glass surface has hydroxyl groups, the monolayer and substrate surface interactions were more effective in hydrophilic glass than in silicon surfaces. A notable difference between the morphologies of the films can be observed. In the case of silicon, incomplete coverage of the monolayer is evidenced by the empty spaces on the smooth silicon surface. For the LB film transferred to FTO glass, complete coverage by the material is observed, consistent with a higher TR value. In both cases, it is possible to observe g-C_3_N_4_ aggregates flakes that were not wholly exfoliated during the sonication process. As a better transfer result was obtained for the FTO-coated glass, this substrate was chosen for the photocatalytic tests using Rh B as a probe molecule and compared with the results of the dispersed material, as seen in the next section.

### 3.4 Photocatalytic Performance of g-C_3_N_4_ LB Films

**Figure 8:**
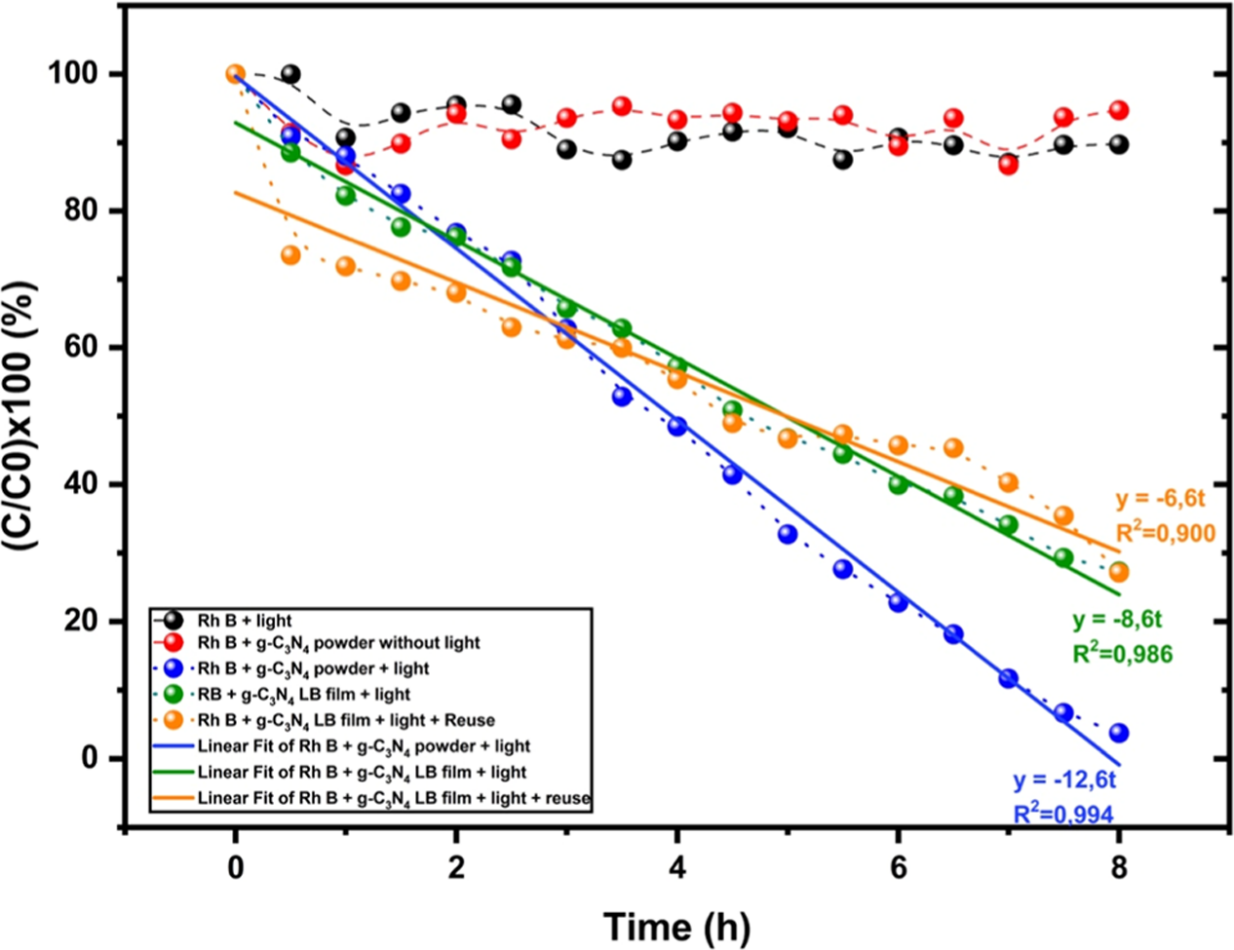
Photocatalytic assay for g-C_3_N_4_ as powder and LB film.

The added value of our LB approach also relies on applying our thin films and their performances as photocatalysts. Here, we would like to test the g-C_3_N_4_ thin film’s photocatalytic properties in the degradation of the Rh B probe molecule, as shown in the set of experimental results displayed in Figure 8. The corresponding UV–vis radiation absorption spectra of Rh B under photodegradation by g-C_3_N_4_ under different experimental conditions are presented in the Supporting Information file, Figures S1–S5.

As we can see, the Rh B probe molecule is not spontaneously degraded in the media, and the concentration remains stable in the presence of g-C_3_N_4_ in the dark (Figure 8, black spheres). Also, when g-C_3_N_4_ is added to the reactor, no significant decrease of the Rh B concentration with time is observed in the absence of xenon lamp light, which corresponds to the expected behavior of g-C_3_N_4_ as a photocatalyst (Figure 8, red spheres). The variations we can observe between 90% and 100% of Rh B concentration are primary artifacts since the sampling method relies on a tiny volume (200 μL aliquot) pulled out of the media and entails more uncertainty on the measurements by UV–vis. On the other hand, a significant concentration decrease is visible and can be clearly interpreted as Rh B degradation observed in the case of Rh B + g-C_3_N_4_ under illumination. The Rh B probe molecule is almost totally degraded within 8 h after starting the experiments (Figure 8, blue spheres).

Figure 8 also presents the curves of degradation of the Rh B probe molecule in contact with the g-C_3_N_4_ LB films under and without illumination with xenon lamp illumination.

In the case of LB thin films of g-C_3_N_4_, the Rh B concentration remains ∼27% within the same timeline (8 h) (Figure 8, green spheres), including for the LB thin films with reuse, 24 h after the first experiment (Figure 8, orange spheres). We repeated the Rh B degradation experiment using the same LB film, which, after being removed from the reactor, was rinsed, air-dried, and left to rest for 24 h. Remarkably, the reused film retained its catalytic activity, achieving approximately 73% degradation of the Rh B molecules despite prior usage.

If we perform a simple chemical kinetic analysis, we will see an apparent 0-order reaction for Rh B degradation for g-C_3_N_4_ powder and LB film. This means that the kinetic law for the photocatalysis reaction is independent of the reactant concentration and leads to a linear relation between the Rh B concentration and time. The kinetic law in the case of an apparent 0 order reaction is *v* = *k*[*C*]^0^ = −d[*C*]/d*t* = *k*, with *v* is the reaction velocity, *C* the remaining concentration of Rh B in the media at a given time, and *k*, the kinetic constant (h^–1^). By integration, we obtain the linear relation *C*(*t*) = −*kt*.

It could be surprising to see an apparent order 0 for the Rh B degradation reaction at first sight since the kinetics should be 0 when one of the reactants (here, Rh B) is almost totally consumed. The g-C_3_N_4_ is not considered a reactant since, as a catalyst, it is not consumed by definition. Zero order reaction generally appears in 2 basic cases: (i) the concentration of the reactant remains practically constant as the reaction progresses. Here, if Rh B concentration is large enough, its concentration will decrease only slightly at the beginning of the reaction. Even if the true order of the reaction is indeed 1 in this case, the apparent order will be zero. We talk about order reaction degenerescence. (ii) The reactant is subject to rapid equilibrium. This second possibility is more plausible in this photocatalysis reaction as the gC_3_N_4_ catalyst can rapidly convert the Rh B molecule and use its active site as much as possible. The linear fit we obtained here with a satisfying correlation is 12.6 h^–1^ for g-C_3_N_4_ powder, 8.6 h^–1^ for g-C_3_N_4_ LB film, and 6.6 h^–1^ for reused g-C_3_N_4_ LB film.

Finally, we can explain that Rh B is not totally degraded by the LB thin films in 8 h of reaction. Although practically the same amount of photocatalyst was present in both systems (approximately 0.0003 g), it can be considered that in the case of the film, the g-C_3_N_4_ sheets have part of their surface in contact with the FTO glass surface, which would block access to one side of the g-C_3_N_4_ sheets. With one side of the sheets being inaccessible, the available area for interaction with light is reduced, which decreases the number of photocatalytic conversions and, consequently, the reaction rate and the amount of dye degraded after 8 h. On the other hand, this discrepancy is expected since the sheets or flakes in the film have one face attached to the substrate, also limiting the dye’s access to photocatalytic sites on the 2D material’s surface. Nonetheless, although the kinetic constant is slightly lower for the reused film (8.6 h^–1^ compared to 6.6 h^–1^), the same amount of dye degradation is achieved 24 h after its prior use (73%). Only good adhesion of the g-C_3_N_4_ layers to the glass surface can ensure that the amount of material transferred by the LB technique remains constant throughout the multiple uses of the catalyst film. This result is a relevant demonstration of the added value of the LB technique in producing resistant and active LB thin films of a 2D catalyst for various applications. It also creates opportunities for other applications involving nanostructured semiconductors and chemical conversion systems, such as photocatalytic devices, photovoltaic systems, or energy storage technologies.

## 4 Conclusions

In the present work, the synthesis and characterization of g-C_3_N_4_ were demonstrated. The ultrasonic exfoliation method enabled isolation of individual sheets of the material, as shown by TEM and SEM experiments. The exfoliated material was used to prepare Langmuir monolayers at the air–liquid interface and characterized by π and *V* versus surface area isotherms. BAM experiments also demonstrate the morphological stages of the monolayers at different surface pressures. Distinct film phases were observed and attributed to different aggregate structures and organization of g-C_3_N_4_ sheets on the air–water interface. Under the BAM view, an appropriate surface pressure corresponding to a dense and homogeneous layer of g-C_3_N_4_ was observed at 25 mN·m^–1^. The LB film transfers were performed onto silicon and FTO-coated glass, with the latter showing excellent coverage, which was chosen for photocatalytic assay. The g-C_3_N_4_ as powder and as an LB film were able to degrade 96.3% and 73%, respectively, of the Rh B present in the aqueous medium after 8 h of reaction. After 24 h, the reused LB film maintained its photocatalytic activity, degrading 73% of the probe molecule. This evidence suggests that the transferred film demonstrates adequate adhesion to the substrate, enabling multiple uses when immersed in an aqueous solution. The results open opportunities for applications involving LB films of g-C_3_N_4_ for photocatalytic, photovoltaic, and other chemical conversion devices.

## Supporting Information Available

Additional experimental results and action spectrum (wavelength dependence) of the Rh B photodegradation process (PDF)
