# Supplementary material for: Graphitic Carbon Nitride: Synthesis and Characterization, Monolayer at the Air–Water Interface, Langmuir–Blodgett Films, and Its Photocatalytic Performance
Source: ACS Omega. 2025 Apr 15;10(16):17024–32. doi: 10.1021/acsomega.5c02295 (PMC12044558; doi:10.1021/acsomega.5c02295)
Supplement: Supplementary file 1 — ao5c02295_si_001.pdf [file ao5c02295_si_001.pdf]

Graphitic Carbon Nitride: Synthesis and Characterization, Monolayer at the Air-Water Interface, Langmuir-Blodgett Films and Its Photocatalytic Performance.

*Éverton Wilker A. Almeida[1], Claire M. C. Dazon[1], Mariandry D. V. R. Rodriguez[1], Thatyane M. Nobre[2], Márcio César Pereira[1] and Douglas S. Monteiro[1] **

[1]Institute of Science, Engineering and Technology, Federal University of Jequitinhonha and Mucuri Valleys, Teófilo Otoni, Minas Gerais, 39803-371, Brazil.

[2]Institute of Physics of São Carlos, University of São Paulo, São Carlos, São Paulo, 13560-970, Brazil

Corresponding Author

Douglas Santos Monteiro

Institute of Science, Engineering and Technology, Federal University of Jequitinhonha and Mucuri Valleys, Teófilo Otoni, Minas Gerais, 39803-371, Brazil.

douglas@ufvjm.edu.br

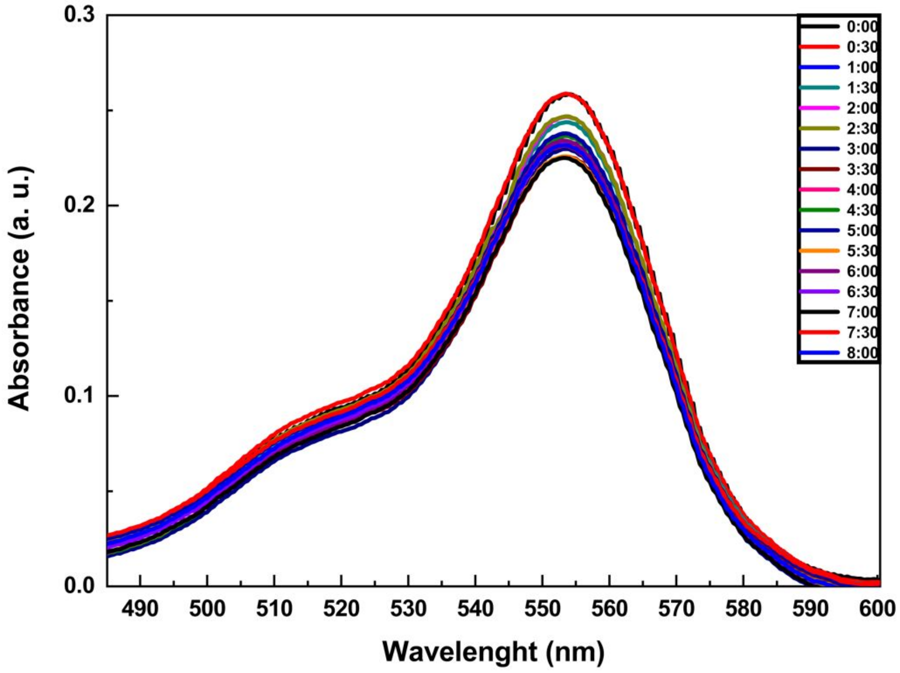

Figure S1 - Rhodamine B photodegradation in presence of light without g-C_3N_4.

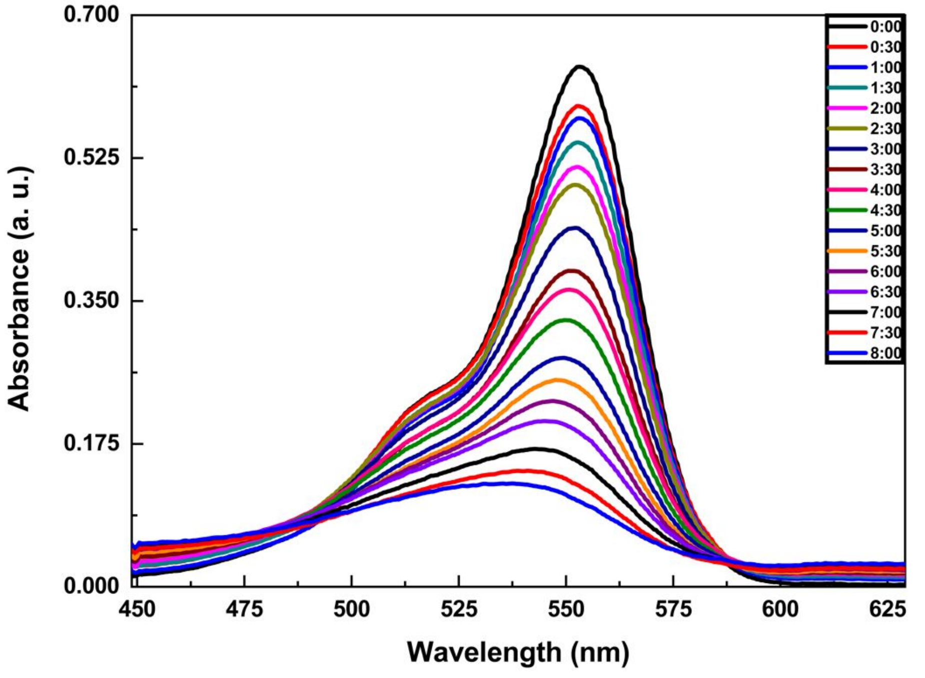

Figure S2 - Rhodamine B photodegradation in presence of light with g-C_3N_4 as powder.

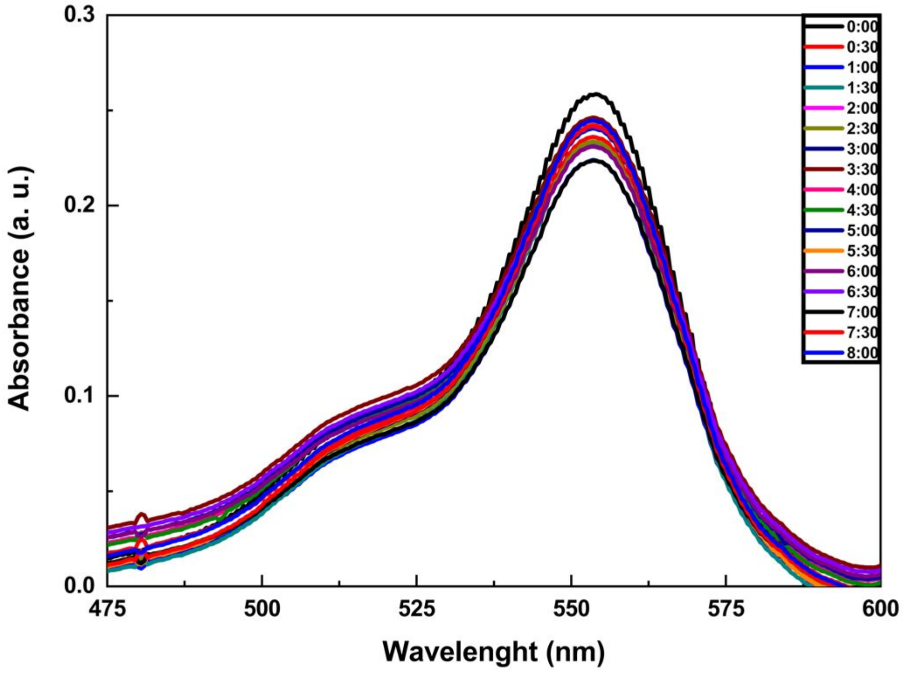

Figure S3 - Rhodamine B photodegradation in presence g-C_3N_4 as LB film without light.

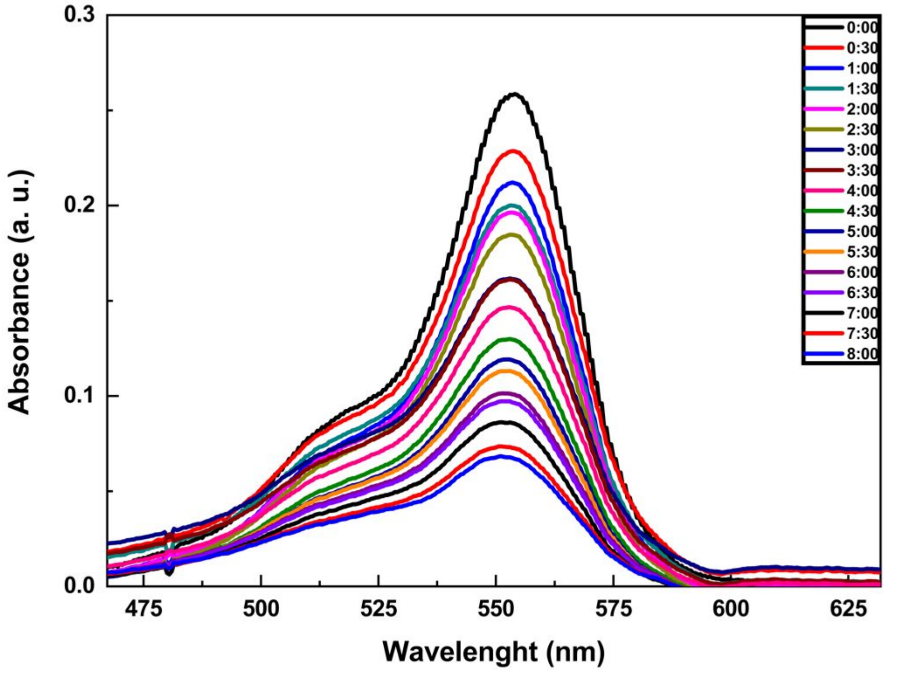

Figure S4 - Rhodamine B photodegradation in presence g-C_3N_4 as LB film with light.

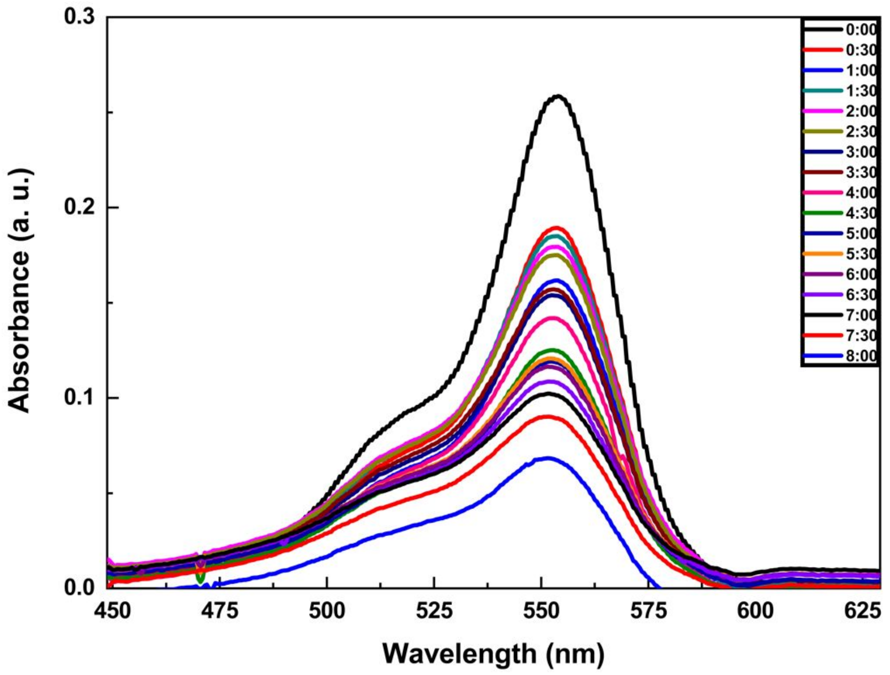

Figure S5 - Rhodamine B photodegradation in presence g-C_3N_4 as LB film with light – second use.
